# Citation Network Analysis of Nurse Staffing Research from the Past Two Decades: 2000–2022

**DOI:** 10.3390/healthcare11233050

**Published:** 2023-11-27

**Authors:** Noriko Morioka, Masanao Ochi, Suguru Okubo, Mutsuko Moriwaki, Kenshi Hayashida, Ichiro Sakata, Masayo Kashiwagi

**Affiliations:** 1 Graduate School of Health Care Sciences, Tokyo Medical and Dental University, Bunkyo-ku, Tokyo 113-8510, Japan; kashiwagi.fnls@tmd.ac.jp; 2 Graduate School of Engineering, University of Tokyo, Bunkyo-Ku, Tokyo 113-8656, Japan; ochi@ipr-ctr.t.u-tokyo.ac.jp (M.O.); isakata@ipr-ctr.t.u-tokyo.ac.jp (I.S.); 3 Institute of Ars Vivendi, Ritsumeikan University, Kyoto 603-8577, Japan; suguruokubo@gmail.com; 4 Quality Management Center Medical Hospital, Tokyo Medical and Dental University, Bunkyo-ku, Tokyo 113-8510, Japan; mmoriwaki.qmc@tmd.ac.jp; 5 Department of Medical Informatics and Management, University Hospital, University of Occupational and Environmental Health, Fukuoka 807-8555, Japan; kenshi@clnc.uoeh-u.ac.jp

**Keywords:** nurse staffing, patient safety, citation analysis, bibliometric analysis, nursing administration research

## Abstract

Studies have indicated that higher numbers of nurses regarding staffing ensure patient safety and a better practice environment. Using citation analysis, this study visualizes the landscape of nurse staffing research over the last two decades to show the overall publication trends, major contributors, and main research topics. We extracted bibliometric information from PubMed from January 2000 to September 2022. After clustering the network, we analyzed each cluster’s characteristics by keyword. A total of 2167 papers were considered for analysis, and 14 clusters were created. The analysis showed that the number of papers published per year has been increasing. Researchers from the US, the UK, Canada, Australia, and Belgium have led this field. As the main clusters in nurse staffing research during the past two decades, the following five research settings were identified: nurse outcome and patient outcome research in acute care hospitals, nurse staffing mandate evaluation research, nursing home research, and school nurse research. The first three clusters accounted for more than 80% of the total number of published papers, and this ratio has not changed in the past 20 years. To further develop nurse staffing research globally, evidence from other geographic areas, such as African and Asian countries, and from long-term care or community settings is necessary.

## 1. Background

Worldwide, approximately 20 million nursing staff, accounting for more than 70% of healthcare professionals, provide patient care on the front lines 24 h a day, 365 days a year [1]. The efficient and effective allocation of nursing staff is a major component of establishing a quality healthcare system. Since early 2000, in the context of developing indicators for quality assurance, the nurse-to-patient ratio and skill mix have been considered as some of the nursing sensitivity indicators [2]. Several studies on appropriate nurse staffing have been conducted since the landmark studies of the early 2000s [3,4]. Analyses of these findings from systematic and umbrella reviews have shown robust evidence for an association between higher nursing staffing and a shorter length of stay, increased patient satisfaction, improved quality of nursing care, fewer readmissions, and reduced in-hospital mortality [5,6,7,8,9,10,11].

Concurrently, to ensure safe nurse staffing, several countries and states have introduced legislation or financial incentives related to nurse staffing ratios in hospitals and other facilities. California was the first US state to enact a minimum nurse-to-patient staffing ratio law in 2004 [12,13]. The state currently requires a minimum ratio of one nurse to five patients in medical–surgical units and one nurse to two patients in critical care units. Massachusetts, USA, requires a 1:1 nurse-to-patient ratio in ICUs. The New York State enacted the “Safe Staffing for Quality Care Act” for acute care facilities and nursing homes in 2021. A roughly 2:1 patient-to-nurse ratio in intensive care units or nursing homes requires facilities to meet a minimum daily average of three and a half hours of nursing care per resident [14]. In Australia, Victoria [15] and Queensland [16] have established regulations and laws regarding the minimum required nurse-to-patient ratio in hospitals, with one nurse to four patients for the day shift and one nurse to seven patients for the night shift. Japan [17] and South Korea [18] have also introduced nurse staffing levels into their financial schemes as patient-to-nurse ratios differentiate the reimbursement of nursing fees. In Japan, there are four categories of nurse staffing in hospitals as follows: the 7:1 patient-to-nurse ratio, 10:1, 13:1, and 15:1. In South Korea, nursing fees are determined by the hospital type and combination of patient-to-registered nurse ratios and patient-to-nursing assistant or ward staff ratios [17]. The UK does not have legal nurse staffing mandates [19]; however, the National Institute for Health and Care Excellence has introduced guidelines for safe nurse staffing levels [20].

To improve nurse staffing policies, it is important to conduct policy evaluations of these regulations in various countries and/or states. In countries without legislation, it is necessary to undertake research on safe nursing placement that captures the characteristics of the country’s healthcare system. However, a geographic bias has been indicated in previous research, which has mostly presented evidence from high-income countries [21,22]. In addition, nurses are active in both hospitals and other areas [1], and there is a lack of evidence and regulations on safe nurse staffing in long-term care settings [23,24] and community settings [25,26]. Appropriate nursing assignments are important not only in acute care settings in acute care hospitals but also in long-term care settings and community settings.

Recently, with the advancement of language processing technology, “bibliometric (or citation network) analysis”, which analyzes bibliographic information, has become increasingly useful in showing the landscape and identifying the major topics of a health service research [27,28]. In nursing research, the themes of “magnet hospital” [29], “nursing student education” [30], or “nurse resilience” [31] have been used to visualize research trends and issues. Regarding nurse staffing research, studies have synthesized the evidence using systematic and umbrella reviews that focus on specific settings and outcomes, for example, intensive care units [9] and mortality in acute care hospitals [6,8,32], and study designs, such as longitudinal studies [10]. As the research on nurse staffing has grown enormously, it has become difficult for traditional manual literature review methods to provide a bird’s-eye view of the entire research area and its trends and hot spots. Bibliometric (or citation network) analysis methods can contribute to uncovering research gaps within nurse staffing research that have not yet been identified and research that remains untapped. Identifying this research gap can ultimately contribute to developing healthcare systems that benefit patients and nurses worldwide.

Consequently, this study aimed to visualize the landscape of nurse staffing research over the past two decades. It used citation analysis to show the overall trends in the number of publications and major contributors and reveal the main research topics and the trends in these topics based on similarities in citation relationships.

## 2. Methods Section

### 2.1. Design and Data Collection

A citation network analysis was conducted. We extracted bibliometric information from PubMed. The search strategy was “nurse staffing” [Title/Abstract] OR “nurse workload” [Title/Abstract] OR “nurse workforce” [Title/Abstract] OR “nurse to patient” [Title/Abstract] OR “patient to nurse” [Title/Abstract] OR “nurse to bed” [Title/Abstract]. We set the publication limit from 1 January 2000 to 30 September 2022 (Appendix A Table A1).

In the search process, unlike traditional literature review methods, all the literature that met the above search strategy was included in the citation analysis. No limitation was imposed on the study setting. Nursing positions or titles were included, ranging from general nursing staff to advanced practice nurses. We also included a broad range of the literature with titles or abstracts in English, even if the main text was in a different language.

### 2.2. Analysis

#### 2.2.1. Citation Network Analysis

We used the “academic-landscape system” at the University of Tokyo, Japan (https://academic-landscape.com/page/about?next=/ accessed on 1 October 2022) (Copyright © 2010–2013 Innovation Policy Research Center, The University of Tokyo.) for the analysis. This system was developed to visualize citation network analyses through the following procedures: (1) retrieving data, (2) determining the maximum connected component and network clustering, and (3) visualization [33]. We performed citation analysis by combining direct citation relationships (when a paper directly cites other papers and analyzing these citation relationships helps us understand the direct connections between papers), co-citation relationships (when multiple papers cite the same other paper and these papers are in a co-citation relationship), bibliographic coupling (when using the same references in their citations, the similarity between papers can be assessed through bibliographic coupling), weighting functions (when representing relationships between papers, assigning weights to each relationship is often used, which allows the importance of different relationships to be considered), keyword similarity (cosine similarity) (by treating keywords in papers as vector representations and calculating the cosine similarity between keyword vectors, the similarity in the content between papers can be evaluated), and modularity maximization (a method that uses the network theory to identify modules or communities within a network) [34,35] In the clustering process, papers that have no direct citation relationship with other papers are automatically excluded from the analysis.

**Figure A1:**
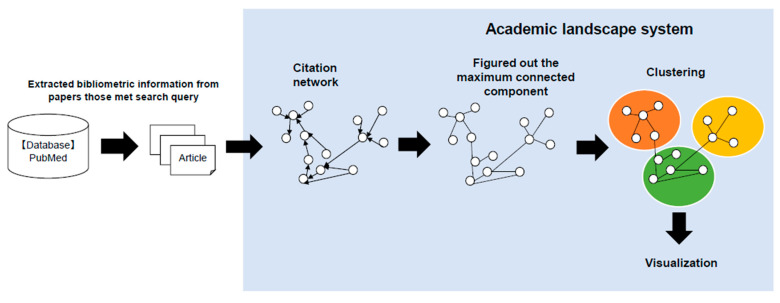
Overview of the citation analysis procedure.

The size of the clusters indicates the number of papers included, and the spatial distance between clusters indicates the similarity of the content. Clusters that are far apart in spatial distance indicate independent research topics with little citation relationship. More details of the analysis are described in Kajikawa et al. [33,36]. The schematic analysis procedure is illustrated in Appendix A, Figure A1.

After clustering the network, we analyzed each cluster’s characteristics by the keywords with higher scores of term frequency-inversed cluster frequency (TF-ICF), titles, and abstracts in papers that were frequently cited by other papers in the cluster in which they were published. TF-ICF is a vector representation of natural language and is an effective method for extracting important words that characterize a cluster. The following equations calculated TF-ICF:

TF-ICF = TF × ICF

where

TF = the term (keyword) counts in the cluster/total word counts in the cluster,

ICF = log[1 − (the total number of clusters/number of cluster including the term)]

The TF-ICF scores were higher for words that occurred more frequently only in the cluster. A higher TF-ICF score indicates more importance regarding the word that characterizes the cluster.

#### 2.2.2. Analysis of Publication Trends, Authors, and Journals

We also carried out a trend analysis of the number of publications, authors, and journals for the articles included in our analysis. For the trend analysis of the number of publications, we visualised each cluster’s annual trends and showed each cluster’s rise and fall over the past 20 years. The affiliations and countries of the top 20 authors, in terms of the number of publications, were listed to reveal the geographical distribution of the nurse staffing studies. The top 20 journals in terms of the number of publications were listed to identify which areas of medicine, nursing, and public health more likely covered the nurse staffing issue.

## 3. Results

### 3.1. Global Publication Trends from 2000 to 2022

**Figure 1:**
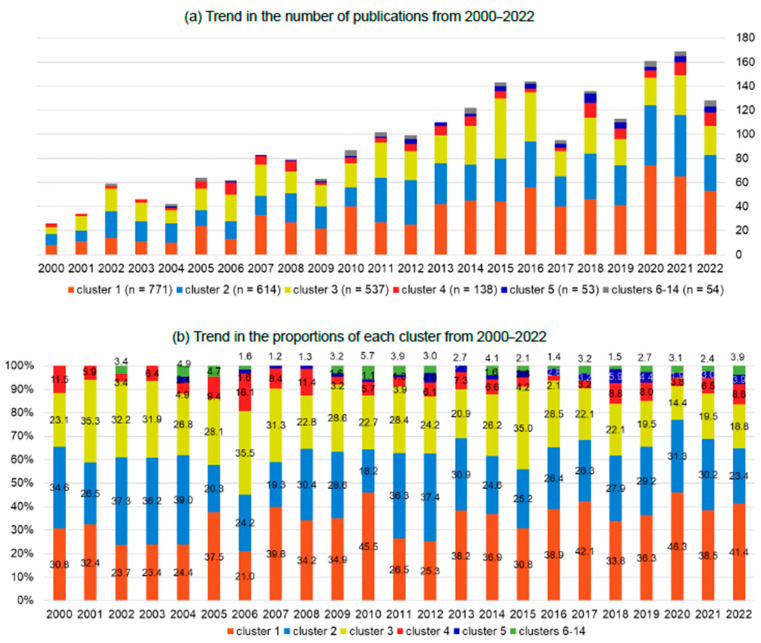
Trends in the number of articles published from 2000 to 2022 (n = 2167). Cluster #1 was titled “nurse outcome research in acute care hospitals”, Cluster #2 was titled “patient outcome research in acute care hospitals”, Cluster #3 was titled “nurse staffing mandate evaluation research”, Cluster #4 was titled “nursing home research”, Cluster #5 was titled “school nurse research”, and Clusters #6–14 are clusters that are small in number and were not included in the top five main clusters. (**a**) Trends in the number of publications from 2000 to 2022; (**b**) Trends in the proportions of each cluster from 2000 to 2022.

Of the 2308 articles returned by the search strategy, 14 clusters were formed from 2167 papers based on citation relationships. In total, 141 articles were automatically dropped during the clustering process because they had no citation relationship with other articles. The overall number of papers published per year has been on the rise, starting with 26 per year in 2000 and gradually increasing to more than 100 per year in 2011 and more than 160 per year after 2020 (Figure 1a).

### 3.2. Analysis of Authors and Journals

Table 1 shows the top 20 most productive authors and their most recent affiliations and countries. Nurse staffing articles were mainly written by authors in the USA, UK, Canada, Australia, and Belgium. The top three researchers who published over 40 papers were Dr. Linda Aiken from the University of Pennsylvania, USA; Dr. Douglas M. Sloane from the University of Pennsylvania, USA; and Dr. Peter Griffiths from the University of Southampton, UK.

Table 2 shows the top 20 journals in terms of their number of publications related to nurse staffing. In descending order, the most publications were in the Journal of Nursing Administration, Journal of Nursing Management, and International Journal of Nursing Studies, which published over 80 related articles each. Some journals in the medical field, but not specifically the nursing field, were also ranked in the top 20, including Medical Care, Health Services Research, Health Affairs, BMJ Open, and the Journal of the American Geriatrics Society.

### 3.3. The Clustered Network Map of Co-Cited References

**Figure 2:**
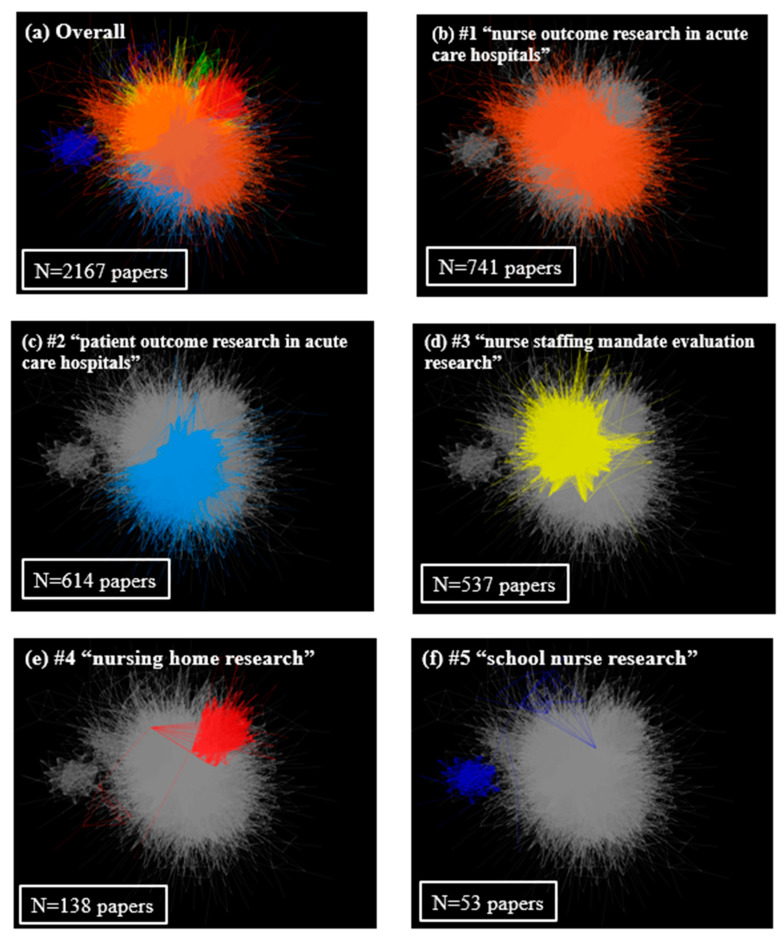
Visualization of the citation network of the top 5 clusters. Cluster #1 was titled “nurse outcome research in acute care hospitals”, Cluster #2 was titled “patient outcome research in acute care hospitals”, Cluster #3 was titled “nurse staffing mandate evaluation research”, Cluster #4 was titled “nursing home research”, and Cluster #5 was titled “school nurse research”. The size of the clusters indicates the number of papers included, and the spatial distance between clusters indicates the similarity of the content. Clusters that are far apart in spatial distance indicate independent research topics with a limited citation relationship.

Out of the 14 clusters, the top five clusters comprised 97.5%. The 6th to 14th clusters, thus, only consisted of a total of 54 papers (2.5%). Therefore, in this paper, we discuss only the top five clusters, which included a level of papers that could be interpreted (Figure 2). Cluster #1 was titled “nurse outcome research in acute care hospitals”, Cluster #2 was titled “patient outcome research in acute care hospitals”, Cluster #3 was titled “nurse staffing mandate evaluation research”, Cluster #4 was titled “nursing home research”, and Cluster #5 was titled “school nurse research”. Over the past 20 years, the proportions of each cluster have not changed (Figure 1b).

Cluster #1, “nurse outcome research in acute care hospitals”, was the largest, with 771 articles. “ICU” (0.00171), “workload” (0.00075), “environment” (0.00061), “quality” (0.00050), and “outcome” (0.00045) were identified as representative key terms with high TF-ICFs (Table 3 and Appendix A Table A2). In this cluster, the papers mainly focused on the association between nurse staffing and nurses’ reporting on the quality of care and nurse outcomes (burnout, intent to stay in their role, satisfaction) in critical care or acute care hospitals (Table 3). The annual number of publications showed an increase overall, although there have been various increases and decreases. This cluster represents an annual share of 30–40% (Figure 1). The annual numbers were around 10 articles from 2000 to 2006, 20–40 articles from 2007 to 2012, and 40–70 articles from 2013 to 2022.

Cluster #2 was titled “patient outcome research in acute care hospitals” and comprised 614 articles. “Mortality” (0.00127), “patient outcome” (0.00104), “hospital” (0.00103), “fall” (0.00078), “surgical” (0.00066), and “readmission” (0.00062) were identified as key terms with high TF-ICFs (Table 3 and Appendix A Table A2). In this cluster, the papers mainly focused on the association between patient outcomes (mortality, length of hospital stay, fall, readmission) in acute care hospitals. Similar to Cluster #1, the annual number of publications in this cluster showed an increase overall and comprised an annual share of 20–37% (Figure 1). The annual numbers were 10–20 articles from 2000 to 2010; since 2011, the annual numbers almost doubled to around 40 articles.

Cluster #3, “nurse staffing mandate evaluation research”, comprised 537 articles. “Staffing level” (0.00225), “patient outcome” (0.00084), “registered nurse” (0.00064), “California” (0.00063), “policy” (0.00063), and “cost” (0.00058) were identified as key terms with high TF-ICFs (Table 3 and Appendix A Table A2). The papers in this cluster focused on the effect of nurse staffing mandates on patient and nurse outcomes, particularly on California’s mandate (Table 3). The annual number of publications in this cluster was around 20 articles from 2000 to 2022, representing a 14–35% annual share (Figure 1). There was an uptrend (n = 50 in 2015) in the four years from 2014 to 2018.

Cluster #4, “nursing home research”, comprised 138 articles. “Nursing home” (0.00728), “resident” (0.00433), “facility” (0.00154), “quality” (0.00127), “medicare” (0.00093), “rating” (0.00076), and “star” (0.00072) were identified as key terms with high TF-ICFs (Table 3 and Appendix A Table A2). In this cluster, the papers mainly focused on the association between nurse staffing and resident outcomes in line with policy changes in the Medicare system in the US, such as the Nursing Home Compare program and star ratings (Table 3). The annual number of publications ranged from 2 to 12, comprising approximately a 10% annual share. There were three uptrends from 2005 to 2008 (10 articles in 2006), 2012 to 2015 (8 articles in 2014 and 2015), and 2018 and 2022 (an average of 10 articles) (Figure 1).

Cluster #5, the “school nurse research” cluster, comprised 53 articles. “School nurse” (0.01518), “school” (0.00933), “student” (0.00395), “mental” (0.00238), “school nurse workload” (0.00231), “asthma” (0.00130), and “epinephrine” (0.00111) were identified as key terms with high IFICFs (Table 3 and Appendix A Table A2). In this cluster, the papers mainly focused on school nurse workload and the association between school nurse staffing and student outcomes in the US (Table 3). The annual number of publications was zero or one from 2000 to 2011; thereafter, fewer than five articles were published annually, except for 2008, when eight were published (Figure 1).

In terms of spatial location, Cluster #1 and Cluster #2, as well as Cluster #1 and Cluster #3, were found to be in close spatial distance and highly similar. Conversely, Clusters #4 and #5 were far from the other clusters and, thus, represented independent research topics (Figure 2).

## 4. Discussion

To the best of our knowledge, this study is the first to capture the landscape of articles on nurse staffing from 2000 to 2022 using a citation network analysis. In terms of the overall publication trends, we found that nurse staffing research has expanded, led by US and UK researchers. We also found that nurse staffing research can be divided into five main clusters according to the co-citation relationship: #1 “nurse outcome research in acute care hospitals”, #2 “patient outcomes research in acute care hospitals”, #3 “nurse staffing mandate evaluation research”, #4 “nursing home research”, and #5 “school nurse research”.

### 4.1. Overall Publication Trends and Leading Authors and Journals from 2000 to 2022

The overall trend of annual publications has been rising, indicating that the nurse staffing research field has been growing. However, it is suggested that more robust evidence on new adequate nurse staffing methods is needed, for instance, flexible nurse staffing methods beyond the use of ratios [49], shift work, working time [50,51], and nurse allocation methodology [52,53]. Thus, the theme of nurse staffing should continue to be a focus in the future.

Researchers from the US, the UK, Canada, Australia, and Belgium have led this field. This finding is consistent with the findings of a previous study focusing on the overall trend of high-impact nursing research papers, which are defined as papers ranked in the top 10% of citation frequency [30]. Among the top 20 contributors, the most prolific can be divided into the following three groups: the first includes Linda H. Aiken and Douglas M. Sloane at the University of Pennsylvania, US; the second includes Peter Griffiths and Jane Ball at the University of Southampton, UK; and the third includes Joan Spetz and Charlene Harrington at the University of California system, US. As for the first group, the University of Pennsylvania was also ranked number one in high-impact nursing research [54]; researchers at the University of Pennsylvania are also the major contributors to, and leaders in, the magnet hospital research [29] and the huge international nurse staffing research group “RN4CAST” was launched in 2007 [55,56]. As for the second group, Griffiths et al. (2016b) from the University of Southampton contributed to the adequate nurse staffing project at the National Institute for Health and Care Excellence in England, leading to the creation of nurse staffing policies in England [20,57]. The third group, Spetz et al. (2004, 2009) from the University of California, led an evaluation of the California nurse staffing mandate and nurse workforce research.

As for journals in which nurse staffing research is published, nursing administration journals are ranked highly. The International Journal of Nursing Studies, which has a broad scope across the nursing field, and other high-impact journals within the broader medical and health service research field also ranked highly, including Medical Care, Health Services Research, Health Affairs, BMJ Open, and the Journal of the American Geriatrics Society. Higher attention to nurse staffing in nursing administration, health policy, and health service research is indicated. However, the publication trends and leading authors and journals over the past two decades demonstrate that there is still limited knowledge and evidence on adequate nurse staffing from other countries, such as low-middle income counties and/or Asian regions with different nurse staffing regulation systems within their healthcare systems.

### 4.2. Cluster by Citation Network Analysis

Five main clusters in nurse staffing research over the past two decades were identified in this study: #1 “nurse outcome research in acute care hospitals”, #2 “patient outcome research in acute care hospitals”, #3 “nurse staffing mandate evaluation research”, #4 “nursing home research”, and #5 “school nurse research”. The first three clusters accounted for more than 80% of the total, and this share has not changed in the past 20 years.

The largest clusters were #1, “nurse outcome research in acute care hospitals”, and #2, “patient outcome research in acute care hospitals”. Although there was no small number of articles that examined both patient and nurse outcomes, those in Cluster #1, “nurse outcome research in acute care hospital”, were relatively more focused on the working environment and nurses’ turnover or retention in relation to the shortage in the nursing workforce.

Clusters #1, “nurse outcome research in acute care hospitals”, and #2, “patient outcome research in acute care hospitals”, were found to be spatially close, indicating the similarity in their context, with the only difference being whether they both focused on nurse outcomes or patient outcomes in nurse staffing research in acute care hospitals. Both clusters examine acute care hospital settings and reflect the trend of quality assurance in healthcare systems, mainly in the US, since the Institute of Medicine’s “To Err is Human” report. To assess the extent to which nursing personnel in acute care hospitals contribute to healthcare quality, patient safety, and a professional and safe work environment, the National Quality Forum developed 15 performance measures for nursing-sensitive care in 2004 [2]. Nursing sensitivity indicators included patient-centered outcomes, nursing-centered outcomes, and system-centered outcomes in nurse staffing and the Practice Environment Scale—Nursing Work Index (PES-NWI) [58]. Our findings indicate an uptrend in Cluster #1, “nurse outcome research in acute care hospitals”, after 2005, which the development of this nursing sensitivity outcome framework may explain. In addition, the uptrend in Cluster #2, “patient outcome research in acute care hospitals”, since 2011, may have been affected by the progress in the international research project RN4CAST [55,56]. Against the backdrop of worldwide nurse workforce shortages, RN4CAST was launched in 2007, coordinated by Walter Sermeus at Katholike Leuven, Belgium, and Linda Aiken at the University of Pennsylvania, to innovate forecasting methods by addressing the volume and quality of nursing staff as well as the quality of patient care. The project targeted 12 European countries, including the USA, Botswana, China, South Africa, and Chile. The findings from this project have been disseminated since 2011; this trend is shown in Figure 2c. Although the RN4CAST has a remarkable output, there are limitations in its cross-sectional design and manner of presenting the mean data, which do not consider the important differences in outcomes, staff characteristics, and care models [59].

Cluster #3 was titled “nurse staffing mandate evaluation research”. The most famous minimum nurse staffing ratio mandate is that of California, US. Since the minimum nurse staffing mandate was introduced in 2004 and became active in 2005, it has been empirically examined as a natural experiment [13,42]. Research articles that examined the expansion of mandates to other states then followed [40]. The spatial distance between Clusters #1, “nurse outcome research in acute care hospitals”, and #3, “nurse staffing mandate evaluation research”, was also close. This may reflect the context in which the nurse staffing mandate in California, US, developed as a California Nursing Association initiative to improve the working environment for nurses and patient safety. Further, there are also some research articles from Australia in which minimum nurse staffing ratio mandates have been introduced [16]. This trend is consistent with a previous systematic review that examined the evaluation of the nurse staffing methodology [60]. Currently, Germany [61], Japan [17], and Korea [18] have reformed nurse staffing mandates and regulations in the payment system, although evaluations of related policy changes are still minimal compared to those in the US and Australia. Although healthcare systems differ from country to country, it is important to share knowledge about the various safe nurse staffing regulations, including laws, payment systems, and guidelines for nursing staffing, and their effects worldwide to build a better nursing delivery system.

In Cluster #4, “nursing home research”, most articles were related to policy changes in Medicare Medicaid certification made by the Centers for Medicare Medicaid Service (CMS) in the US [62]. The Nursing Home Compare website was launched in 1998 for quality assurance in nursing homes and has since undergone several revisions. The Nursing Home Compare program presents the following five categories of information: inspection results, including deficiencies from Medicare; certification surveys and complaint investigations; facility characteristics; nursing home staffing levels; and quality measures, which provide information on the clinical and physical characteristics of a nursing home’s residents. This information is retrieved from the Online Survey Certification and Reporting (OSCAR) data and the Long-Term Care Minimum Data Set (MDS) Repository. In 2008, CMS launched the Five-Star Quality Rating System to help consumers, their families, and caregivers compare nursing homes more easily and identify areas about which they wanted to ask questions [63]. In addition, since 2002, nursing home pay-for-performance programs, based on the quality of the chronic care delivered and using financial incentives tied to Medicaid or Medicare payment, have been implemented in some states and CMSs [44]. Over the past two decades, approximately 10 papers have been published in this cluster annually. Understanding nurse staffing and its association with resident and nurse outcomes in long-term care settings is necessary. In addition, the importance of research in long-term care settings is even greater for both Asian countries with already super-aged societies and Asian and African countries that could become aged societies in the relatively near future.

Cluster #5, “school nurse research”, was a relatively newly developed area of research. The annual number of papers was over five for the first time in 2018, and the total amount of research in this cluster is somewhat limited compared to research in acute care hospital settings. School nurses take on the role of case managers, bringing providers, families, and schools together to support students’ health; as a result, better attendance and academic success are gained [64,65]. Some states in the US recommend one school nurse for every 750 students in the healthy student population, a ratio of 1:225 for student populations requiring daily professional nursing services, a ratio of 1:125 for student populations with complex healthcare needs, and a ratio of 1:1 for individual students requiring daily, continuous professional nursing services [66]. However, the National Association of School Nurses stated that the workload of school nurses has been expanding in line with an increase in children with mental health issues and those requiring special medical treatment [66]. More evidence is necessary to ensure adequate and safe nurse staffing in school settings.

### 4.3. Limitations

This study has some limitations. The first relates to the search database. The WOS database is widely used in bibliometric analysis; however, we used PubMed because we could only access the current trends via WOS from 2000 to 2011 due to funding limitations. However, using PubMed has some benefits. For example, PubMed has the advantages of being the best database in the medical field, having an optimal update frequency, and including early online articles [67]. Future studies should extend the search to WOS and other databases. Second, we could not include articles in which the title, abstract, and text were in a language other than English. Thus, we might have overestimated the geographic bias of the evidence on nurse staffing. Nevertheless, this citation analysis provides a more comprehensive picture of nurse staffing research in that, even if the text was in another language, it was included as long as some parts of the article, such as the title and abstract, were in English. Thus, this study included studies from the literature that have been excluded from traditional literature reviews. Third, since the method used in this study analyzes direct citation networks, the results may be biased due to the issue that papers already cited in other works in the literature are more likely to be cited in new papers. In recent years, it has been noted that evaluating researchers using the citation matrix is invalid as it depends on citation and publication counts [63]. Alternative indexes considering co-author contributions and publication age beyond the sole publication count have been developed [68]. Fourth, although we revealed a landscape of over 2000 nurse staffing articles from the viewpoint of the citation network, we could not evaluate the research quality of each article in the same manner as a systematic review. However, it is crucial to capture the latest research trends in real time from the rapidly growing academic literature. We believe that sharing the results of our analysis with the nursing community, including researchers, could provide objective evidence to help determine this community’s future direction.

## 5. Conclusions

Using citation analysis, nurse staffing research over the past two decades formed five major clusters, depending on the study setting and outcomes focused on. This landscape of over 2000 nurse staffing articles revealed that evidence regarding long-term care settings and/or those from other geographic areas is still small compared to those in acute care settings from the US or UK. To ensure the safety of patients and nurses in all practice settings and locations, diverse geographic and setting knowledge is essential.

## Figures and Tables

**Table 1:** Affiliations and countries of the top 20 authors.

Author	Number of Articles	Affiliation	Country	
Linda H. Aiken	95	University of Pennsylvania	USA	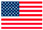
Douglas M. Sloane	51	University of Pennsylvania	USA	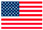
Peter Griffiths	44	University of Southampton	UK	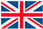
Matthew D. McHugh	36	University of Pennsylvania	USA	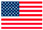
Joanne Spetz	27	University of California, San Francisco	USA	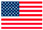
Sean P. Clarke	27	McGill University, Quebec	Canada	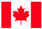
Charlene Harrington	25	University of California, San Francisco	USA	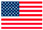
Walter Sermeus	23	KU Leuven-University of Leuven	Belgium	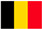
Eileen T. Lake	22	University of Pennsylvania	USA	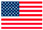
Christine Duffield	21	University of Technology Sydney/Edith Cowan University	Australia	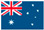
Jeannie P. Cimiotti	20	Emory University, Georgia	USA	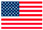
Kathleen Rice Simpson	19	Mercy Hospital, Missouri	USA	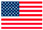
Barbara A. Mark	19	University of North Carolina at Chapel Hill	USA	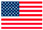
Jane Ball	18	University of Southampton	UK	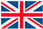
Peter I. Buerhaus	18	Montana State University	USA	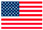
Jack Needleman	18	University of California, Los Angeles, San Francisco	USA	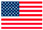
Koen Van den Heede	17	KU Leuven-University of Leuven	Belgium	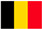
David W. Harless	16	Virginia Commonwealth University	USA	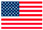
Vincent S. Staggs	15	University of Missouri-Kansas City, Missouri	USA	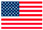
Luk Bruyneel	13	KU Leuven-University of Leuven	Belgium	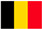
Anne Marie Rafferty	13	King’s College London	UK	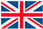

The most recent literature in the PubMed search results was used to identify each author’s affiliation. UK; United Kingdom, USA; United States of America.

**Table 2:** Titles and Journal Impact Factor^TM^ of top 20 journals.

Journal Title	Number of Articles	Journal Impact Factor in 2021
Journal of Nursing Administration	88	1.806
Journal of Nursing Management	85	4.682
International Journal of Nursing Studies	81	6.612
Journal of Advanced Nursing	52	3.057
Nursing Economics	49	1.193
Medical Care	47	3.178
Health Services Research	45	3.734
Journal of Clinical Nursing	43	4.423
Nursing Standard	43	no data
Journal of Nursing Scholarship	33	3.928
Policy, Politics & Nursing Practice	33	no data
Journal of Nursing Care Quality	29	1.728
Health Affairs	29	9.048
BMJ Open	23	3.007
International Nursing Review	23	3.384
American Journal of Nursing	22	2.577
MCN: The American Journal of Maternal-Child Nursing	21	1.753
Nursing Research	19	2.364
Nursing Outlook	19	3.315
Nursing Times	19	no data
Journal of the American Geriatrics Society	19	7.538
Modern Healthcare	19	no data

Journal Impact Factor^TM^ was taken from the Web of Science (Clarivate).

**Table 3:** Representative keywords and papers in the top five clusters.

Cluster Name	Keywords (TF-ICF)	Examples of Included Papers
#1 “nurse outcome research in acute care hospital”	ICU (0.00171), workforce (0.00085), nursing care (0.00084), workload (0.00075), work environment (0.00056), missed (0.00056)	[3,37]
#2 “patient outcome research in acute care hospitals”	mortality (0.00127), patient outcome (0.00104), hospital (0.00103), patient (0.00080), fall (0.00078), outcome (0.00077)	[4,38,39]
#3 “nurse staffing mandate evaluation research”	staffing level (0.00225), patient outcome (0.00084), hospital (0.00065), registered nurse (0.00064), California (0.00063), policy (0.00063)	[40,41,42]
#4 “nursing home research”	nursing home (0.00728), resident (0.00433), medicare (0.00105), deficiency (0.00096), Medicaid (0.00093), nursing facility (0.00089)	[43,44,45]
#5 “school nurse research”	school nurse (0.01518), school (0.00933), student (0.00395), mental (0.00238), school nurse workload (0.00231), asthma (0.00130)	[46,47,48]

TF-ICF: term frequency-inverse cluster frequency.

## Data Availability

Publicly available datasets were analysed in this study. All data are provided in the article. Further data can be provided on request.

## Appendix A

**Table A1:** Search strategy.

	Number of Articles That Met the Search Query
“nurse staffing”	26,194
“nurse workload”	8113
“nurse workforce”	18,801
“nurses to patients”	163,885
“nurse to patients”	163,885
“nurse to patient”	163,885
“nurses-to-patients”	163,885
“nurse-to-patients”	163,885
“nurse-to-patient”	414
“patients to nurses”	163,885
“patient to nurses”	163,885
“patient to nurse”	163,885
“patients-to-nurses”	163,885
“patient-to-nurses”	163,885
“patient-to-nurse”	171
“nurses to beds”	2357
“nurse to beds”	2357
“nurse to bed”	7235
“nurses-to-beds”	7235
“nurse-to-beds”	7235
“nurse-to-bed”	39
number of nurses	29,725
“nurse staffing” [Title/Abstract]	1752
“nurse workload” [Title/Abstract]	147
“nurse workforce” [Title/Abstract]	280
“nurses to patients” [Title/Abstract]	0
“nurse to patients” [Title/Abstract]	0
“nurse to patient” [Title/Abstract]	415
“nurses-to-patients” [Title/Abstract]	0
“nurse-to-patients” [Title/Abstract]	0
“nurse-to-patient” [Title/Abstract]	415
“patients to nurses” [Title/Abstract]	0
“patient to nurses” [Title/Abstract]	0
“patient to nurse” [Title/Abstract]	171
“patients-to-nurses” [Title/Abstract]	0
“patient-to-nurses” [Title/Abstract]	0
“patient-to-nurse” [Title/Abstract]	171
“nurses to beds” [Title/Abstract]	0
“nurse to beds” [Title/Abstract]	0
“nurse to bed” [Title/Abstract]	39
“nurses-to-beds” [Title/Abstract]	0
“nurse-to-beds” [Title/Abstract]	0
“nurse-to-bed” [Title/Abstract]	39
“nurse staffing” [Title/Abstract] OR “nurse workload” [Title/Abstract] OR “nurse workforce” [Title/Abstract] OR “nurse to patient” [Title/Abstract] OR “patient to nurse” [Title/Abstract] OR “nurse to bed” [Title/Abstract]	2563

**Table A2:** Details of the TF-ICF score of each keyword in the five main clusters.

Cluster #1							Cluster #2							Cluster #3					
TERM	TFICF	TC	CC	TF	ICF		TERM	TFICF	TC	CC	TF	ICF		TERM	TFICF	TC	CC	TF	ICF
icu	0.00171	652	4	0.00314	0.54407		mortality	0.00127	591	6	0.00344	0.36798		staffing level	0.00225	563	6	0.00611	0.36798
nursing	0.00130	2575	11	0.01239	0.10474		patient outcome	0.00104	398	5	0.00232	0.44716		level	0.00219	830	8	0.00901	0.24304
intensive care	0.00086	597	7	0.00287	0.30103		hospital	0.00103	2654	12	0.01545	0.06695		nurse staffing level	0.00171	352	5	0.00382	0.44716
workforce	0.00085	584	7	0.00281	0.30103		level	0.00089	627	8	0.00365	0.24304		nurse staffing	0.00116	1594	12	0.01730	0.06695
nursing care	0.00084	582	7	0.00280	0.30103		patient	0.00080	4249	13	0.02473	0.03218		staffing	0.00092	2626	13	0.02850	0.03218
workload	0.00075	517	7	0.00249	0.30103		icu	0.00079	250	4	0.00145	0.54407		patient outcome	0.00084	174	5	0.00189	0.44716
intensive care unit	0.00073	413	6	0.00199	0.36798		fall	0.00078	246	4	0.00143	0.54407		outcome	0.00078	493	10	0.00535	0.14613
care unit	0.00069	479	7	0.00231	0.30103		outcome	0.00077	902	10	0.00525	0.14613		relationship	0.00077	237	7	0.00257	0.30103
unit	0.00066	942	10	0.00453	0.14613		nursing	0.00069	1135	11	0.00661	0.10474		nursing	0.00076	665	11	0.00722	0.10474
level	0.00066	562	8	0.00270	0.24304		rate	0.00067	475	8	0.00276	0.24304		registered	0.00065	199	7	0.00216	0.30103
work	0.00063	679	9	0.00327	0.19189		surgical	0.00066	252	5	0.00147	0.44716		hospital	0.00065	889	12	0.00965	0.06695
job	0.00062	429	7	0.00206	0.30103		readmission	0.00062	237	5	0.00138	0.44716		registered nurse	0.00064	195	7	0.00212	0.30103
environment	0.00061	522	8	0.00251	0.24304		unit	0.00060	700	10	0.00407	0.14613		california	0.00063	107	4	0.00116	0.54407
critical care	0.00058	221	4	0.00106	0.54407		cost	0.00056	317	7	0.00184	0.30103		policy	0.00063	157	6	0.00170	0.36798
intensive	0.00057	622	9	0.00299	0.19189		data	0.00055	646	10	0.00376	0.14613		data	0.00060	377	10	0.00409	0.14613
work environment	0.00056	261	5	0.00126	0.44716		ratio	0.00054	887	11	0.00516	0.10474		mortality	0.00059	148	6	0.00161	0.36798
missed	0.00056	259	5	0.00125	0.44716		effect	0.00052	298	7	0.00173	0.30103		cost	0.00058	178	7	0.00193	0.30103
turnover	0.00055	211	4	0.00102	0.54407		ulcer	0.00052	164	4	0.00095	0.54407		staffing and patient	0.00053	73	3	0.00079	0.66901
care	0.00051	3275	13	0.01576	0.03218		associated	0.00051	600	10	0.00349	0.14613		effect	0.00051	156	7	0.00169	0.30103
quality	0.00050	718	10	0.00346	0.14613		surgery	0.00051	195	5	0.00113	0.44716		quality	0.00050	317	10	0.00344	0.14613
critical	0.00049	279	6	0.00134	0.36798		complication	0.00050	158	4	0.00092	0.54407		nurse staffing and patient	0.00049	68	3	0.00074	0.66901
infant	0.00046	175	4	0.00084	0.54407		workload	0.00050	283	7	0.00165	0.30103		unit	0.00048	302	10	0.00328	0.14613
outcome	0.00045	638	10	0.00307	0.14613		risk	0.00047	334	8	0.00194	0.24304		research	0.00045	218	9	0.00237	0.19189
job satisfaction	0.00044	169	4	0.00081	0.54407		pressure ulcer	0.00047	149	4	0.00087	0.54407		skill mix	0.00045	93	5	0.00101	0.44716
data	0.00043	609	10	0.00293	0.14613		odds	0.00046	214	6	0.00125	0.36798		association	0.00043	207	9	0.00225	0.19189
satisfaction	0.00042	291	7	0.00140	0.30103		hospital mortality	0.00045	116	3	0.00068	0.66901		evidence	0.00043	205	9	0.00223	0.19189
registered	0.00042	289	7	0.00139	0.30103		lower	0.00045	257	7	0.00150	0.30103		safety	0.00042	158	8	0.00171	0.24304
mortality	0.00041	234	6	0.00113	0.36798		staffing level	0.00044	205	6	0.00119	0.36798		mix	0.00041	126	7	0.00137	0.30103
decision	0.00035	163	5	0.00078	0.44716		patient ratio	0.00039	352	9	0.00205	0.19189		model	0.00035	169	9	0.00183	0.19189
**Cluster #4**							**Cluster #5**												
**TERM**	**TFICF**	**TC**	**CC**	**TF**	**ICF**		**TERM**	**TFICF**	**TC**	**CC**	**TF**	**ICF**							
nursing home	0.00728	647	5	0.01627	0.44716		school nurse	0.01518	164	1	0.01324	1.14613							
resident	0.00433	385	5	0.00968	0.44716		school	0.00933	314	6	0.02535	0.36798							
home	0.00418	684	8	0.01720	0.24304		student	0.00395	90	4	0.00727	0.54407							
nursing	0.00223	845	11	0.02125	0.10474		school nursing	0.00305	33	1	0.00266	1.14613							
facility	0.00154	252	8	0.00634	0.24304		mental	0.00238	66	5	0.00533	0.44716							
quality	0.00127	345	10	0.00868	0.14613		school nurse workload	0.00231	25	1	0.00202	1.14613							
home resident	0.00116	69	3	0.00174	0.66901		mental health	0.00206	57	5	0.00460	0.44716							
nursing home resident	0.00116	69	3	0.00174	0.66901		pmh	0.00176	19	1	0.00153	1.14613							
medicare	0.00105	77	4	0.00194	0.54407		health	0.00169	200	11	0.01615	0.10474							
level	0.00105	171	8	0.00430	0.24304		workforce	0.00158	65	7	0.00525	0.30103							
deficiency	0.00096	85	5	0.00214	0.44716		school health	0.00157	17	1	0.00137	1.14613							
chain	0.00094	56	3	0.00141	0.66901		workload	0.00146	60	7	0.00484	0.30103							
medicaid	0.00093	83	5	0.00209	0.44716		asthma	0.00130	24	3	0.00194	0.66901							
nursing facility	0.00089	53	3	0.00133	0.66901		epinephrine	0.00111	12	1	0.00097	1.14613							
life care	0.00087	41	2	0.00103	0.84510		psychiatric mental	0.00102	11	1	0.00089	1.14613							
registered	0.00083	110	7	0.00277	0.30103		psychiatric mental health	0.00102	11	1	0.00089	1.14613							
registered nurse	0.00081	107	7	0.00269	0.30103		school setting	0.00102	11	1	0.00089	1.14613							
rating	0.00076	68	5	0.00171	0.44716		adolescent	0.00096	14	2	0.00113	0.84510							
star	0.00072	43	3	0.00108	0.66901		school district	0.00093	10	1	0.00081	1.14613							
hprd	0.00072	34	2	0.00086	0.84510		school nurse staffing	0.00093	10	1	0.00081	1.14613							
staffing level	0.00069	75	6	0.00189	0.36798		public school	0.00093	10	1	0.00081	1.14613							
home quality	0.00066	31	2	0.00078	0.84510		school nurse workforce	0.00093	10	1	0.00081	1.14613							
nursing home quality	0.00066	31	2	0.00078	0.84510		anaphylaxis	0.00093	10	1	0.00081	1.14613							
profit	0.00062	45	4	0.00113	0.54407		allergy	0.00093	10	1	0.00081	1.14613							
state	0.00060	125	9	0.00314	0.19189		hop	0.00093	10	1	0.00081	1.14613							
case	0.00058	77	7	0.00194	0.30103		psychiatric	0.00083	23	5	0.00186	0.44716							
effect	0.00056	74	7	0.00186	0.30103		policy	0.00077	26	6	0.00210	0.36798							
relationship	0.00055	73	7	0.00184	0.30103		academic outcome	0.00074	8	1	0.00065	1.14613							
life	0.00045	60	7	0.00151	0.30103		nursing	0.00068	80	11	0.00646	0.10474							

CC: number of clusters including the term, COVID: coronavirus disease, HPRD: hours per resident day, ICF: log [1 − (total number of clusters/CC)], ICU: intensive care unit, PMH: Psychiatric Mental Health, TC: term (keyword) counts in the cluster, TF-ICF = TF × ICF, WISN: workload indicators of staffing needs.
